# Editorial: Understanding retinal remodeling: Retinal alterations and therapeutic implications

**DOI:** 10.3389/fnana.2023.1191906

**Published:** 2023-04-05

**Authors:** Diego García-Ayuso, Gema Esquiva, Jose R. Hombrebueno

**Affiliations:** ^1^Departamento de Oftalmología, Facultad de Medicina, Universidad de Murcia, and Instituto Murciano de Investigación Biosanitaria Hospital Virgen de la Arrixaca (IMIB-Virgen de la Arrixaca), Murcia, Spain; ^2^Departamento de Óptica, Farmacología y Anatomía, Universidad de Alicante, Alicante, Spain; ^3^Institute of Inflammation and Ageing, College of Medical and Dental Sciences, University of Birmingham, Birmingham, United Kingdom

**Keywords:** photoreceptor, retinal degeneration, retinal remodeling, retinal ganglion cells, neuroprotection

Retinal remodeling refers to a series of progressive pathological changes in the retina that are caused by photoreceptor degeneration (Marc et al., 2003). This is (primarily) triggered by inherited mutations affecting rods, cones, or the retinal pigment epithelium, as well as by environmental stressors, such as light or diet, that combine with genetic risk to cause photoreceptor degeneration (Jones et al., 2003; García-Ayuso et al., 2018a; Trouillet et al., 2018). The process begins with the primary loss of photoreceptors, leading to astrogliosis (Di Pierdomenico et al., 2019) and secondary loss of remaining photoreceptors (Hombrebueno et al., 2010; Di Pierdomenico et al., 2020). This is followed by the progressive remodeling of inner retinal tissue (Pfeiffer et al., 2020), as underpinned by abnormal synaptic rewiring and loss of inner retinal neurons, including ganglion cells (García-Ayuso et al., 2018b), which are responsible for the transmission of visual information to the brain. Therefore, understanding retinal remodeling and investigating new strategies to alleviate this process is essential to improve the success of interventions aimed at protecting vision in photoreceptor degenerations. This Research Topic includes five manuscripts to help understand this challenging process:

Pfeiffer and Jones provide new perspectives on the pathological events that occur in photoreceptor degenerations by focusing on the challenges posed by secondary retinal remodeling, such as microglial activation and migration, Mülller cell gliosis, Müller cell seal formation, protein upregulation, rewiring, and widespread neurodegeneration, in the development of therapies aimed at restoring lost vision. The authors emphasize the complexity of retinal degenerations given their heterogeneity, which imposes a significant obstacle for the common success of therapeutic strategies, as based on gene therapy, optogenetics, photo-switches, cell replacement, bionics or diffusible molecules for neuroprotection. They concluded that a combinatorial approach of interventions alleviating retinal degeneration along with cellular replacement, would be essential for therapeutic success.

Martinez-Galan examined brain cortical changes in adults with severe retinal degeneration, as well as at the preclinical level in the P23H-1 rat model of Retinitis Pigmentosa (RP). By examining the synaptic architecture of the primary visual cortex, they showed that RP significantly decreased the density of dendritic spines and altered their distribution. The most notable changes in the visual cortex occurred after a prolonged period of retinal degeneration, by affecting the presynaptic thalamocortical VGLUT2-immunoreactive terminals and postsynaptic dendritic spines of layer V pyramidal cells. This study shows, for the first time, morphological changes in the visual cortices of RP rodents, involving an excellent preclinical resource to understand how retinal degeneration impacts on the visual cortex and whether it can be alleviated through different therapeutic strategies.

Ziółkowska and Lewczuk examine the mRNA expression profiles of rhodopsin, melanopsin, c-Fos, and Birc5 in the retinas of albino rats under exposure to white and monochromatic light. The results showed that the photoreceptor inner and outer segment measurements were significantly decreased, accompanied by reduced expression of Rho and Opn4 mRNA expression after exposure to blue and green light. However, increased Birc5 and c-fos mRNA expression was observed after exposure to these types of light. These authors conclude that the increased expression of the anti-apoptotic gene Birc5 may be an early response that helps to reduce light-induced retinal damage.

Using an *ex vivo* model of spontaneous neuroretinal degeneration in pig eyes. Puertas-Neyra et al. described the longitudinal changes associated with key molecular pathways that control cellular death. Concomitant with neuroretinal degeneration and astroglial cell stress, they identified an upregulation of major effectors involved in apoptosis (e.g., caspases) and necroptosis (e.g., receptor-interacting protein kinases). Interestingly, such molecular changes are associated with dysregulation of the autophagy machinery, which is critical for neuroretinal homeostasis and is impaired in a plethora of ocular conditions (Hombrebueno et al., 2019), including inherited retinal degenerations (Punzo et al., 2009).

Finally, Reynisson et al. provide further insights into how Müller cell dysfunction may impact neurodegeneration in the inner retina during the remodeling phase. In addition to recent evidence (Pfeiffer et al., 2020) and using *rd1* mice, they quantitatively assess how Müller glia stress in discrete areas (identified by glutamine synthetase loss) is associated with inner retinal neuronal deterioration, particularly to the loss of bipolar and amacrine cell populations from rod- and cone-driven visual pathways. This study strengthens the paradigm that safeguarding Müller glial function is pivotal for neuroprotective strategies aimed at protecting vision in photoreceptor degenerations.

We hope that this Research Topic has helped to clarify certain aspects of retinal remodeling that occur as a secondary consequence of photoreceptor death, thereby facilitating a better understanding of this phenomenon to help maximize interventions by optimizing the most promising therapeutic strategies.

## Author contributions

All authors listed have made a substantial, direct, and intellectual contribution to the work and approved it for publication.
